# Rehabilitation environments: Service users’ perspective

**DOI:** 10.1111/hex.12859

**Published:** 2019-01-10

**Authors:** Maggie Killington, Dean Fyfe, Allan Patching, Paul Habib, Annabel McNamara, Rachael Kay, Venugopal Kochiyil, Maria Crotty

**Affiliations:** ^1^ Southern Adelaide Local Health Network Bedford Park South Australia Australia; ^2^ Flinders University Bedford Park South Australia Australia; ^3^ Central Adelaide Local Health Network Adelaide South Australia Australia; ^4^ Northern Adelaide Local Health Network Adelaide South Australia Australia

**Keywords:** buildings, carers, choices, clinicians, consumers, environments, outside, patients, rehabilitation, well‐being

## Abstract

**Background:**

Design of rehabilitation environments is usually “expert” driven with little consideration given to the perceptions of service users, especially patients and informal carers. There is a need to engage with consumers of services to gain their insights into what design aspects are required to facilitate optimum physical activity, social interaction and psychological responses when they are attempting to overcome their limitations and regain function.

**Research design:**

Qualitative exploratory study.

**Method:**

Interviews were conducted with patients (n = 54) and informal carers (n = 23), and focus groups with rehabilitation staff (n = 90), from the three metropolitan South Australia rehabilitation health services, comprising different building and environmental configurations. Thematic analysis was assisted by the use of NVivo 11 qualitative software, with pooled data from all interviews and focus groups undergoing open, axial and finally selective coding.

**Results:**

Four major themes were identified as follows: (a) choice can be an Illusion in a rehabilitation ward; (b) access to outside areas is a priority and affects well‐being; (c) socialization can be facilitated by the environment; and (d) ward configuration should align with the model of care.

**Discussion and Conclusion:**

Participants who encountered the most restrictive environments accepted their situation until probed to consider alternatives; those who enjoyed the most choice and access to facilities showed the greatest enthusiasm for these affordances. Future architectural designers should therefore consider the perceptions of a wide range of consumers with varying experiences to ensure they understand the complex requirements of patients and that the ward design facilitates the optimum rehabilitation model of care.

## 1 BACKGROUND

There is a paucity of evidence that addresses the consumer perspective of optimum rehabilitation environments that support models of care known to improve the outcomes of patients who have suffered an illness or injury. Pryor^1^ reports that the creation of a rehabilitative milieu for an inpatient setting should consider “external and internal design and location of buildings; adequacy, allocation, usage and availability of space; and location of equipment and facilities”. Current design knowledge reflects on “expert‐driven and expert determined outcomes” with little input from the end users, patients, staff and visitors.^2^ It is important that consumers of facilities are involved in the design to ensure architects and health planners understand the complex requirements at design phase and then in later years to promote consistent and appropriate changes after the design team has completed their task.^2^

General rehabilitation is part of all patient care, including acute care, and involves the prevention, assessment, management and supervision of a person with a disability until that person has attained an adequate and appropriate level of performance.^3^ Therefore, it seems clear that planning such facilities should take note of affordances, defined as “opportunities to engage and to act in well‐learned or instinctive ways”^4^ or “what we choose to do, given the opportunity”.^5^ Although we tend to spend considerable time considering superficial aspects of design like colours and sounds, these “raw sense data” do not provide the more important opportunities that the actual building design can provide: involvement with aspects that are recognizable, and able to be manipulated and used.^6^ The commonly used alternative of television watching used frequently in rehabilitation units defines a diminished environment if there are no other activities offered.

As part of a health reform process commenced in public hospitals in South Australia where rehabilitation services were moved and beds redistributed, we commenced an investigation of consumer preferences prior to service reconfigurations.^7^ This project was collaboration between consumer investigators from the three metropolitan local health networks of Adelaide, South Australia, and researchers from Flinders University and South Australia Department of Health. This collaborative approach has been promoted as a way of ensuring credibility and relevance of health research.^8^, ^9^, ^10^, ^11^

A model of care provides a framework to guide the way in which rehabilitation is delivered and should, where possible, reflect best practice evidence. Recently, in South Australia, a change to the way rehabilitation is delivered was guided by a model of care document which supported re‐development of a sustainable service defined by six key objectives. The model of care defined provision of rehabilitation that is person‐ and family‐centred, safe, effective, accessible, efficient and equitable.^12^

Investigations have been undertaken that compare the effect on patients of sunlight, sound, odour, windows and spatial layout on variables including fear, arousal, anger/aggression, sadness and attentiveness with currently no conclusive findings to guide architectural practice in a hospital environment.^13^

A number of design frameworks have been proposed over the years to help describe and categorize the important aspects of health design.^14^, ^15^, ^16^ Roger Ulrich stressed that rehabilitation environments should move beyond functional efficiency and costs and focus predominately on providing a stress‐free environment, and the way to do this was to design a psychologically supportive environment to help ameliorate the stress of those receiving health care and their significant others.^14^ Gesler, a health geographer, subsequently developed the notion of a “therapeutic landscape concept,” and he suggested that we could categorize this concept into physical (natural and man‐made) environments, social environments and symbolic environments. In addition, Lawton and colleagues (1978, 1983, 1985) and Lichtenberg and colleagues (2000) introduced and subsequently investigated the Theory of Environmental Press that describes the interaction between an older persons’ competencies and the environmental variables (environmental press).^15^, ^17^, ^18^, ^19^ This current study sought clarification from participants to understand how these theories might relate to their experience in rehabilitation facilities in South Australia by developing an interview guide which probed participants to consider these aspects of their environment.

### 1.1 Research aims

To understand patients’ perceptions of how the rehabilitation environment enhanced and/or reduced the quality of their rehabilitation journey.

To understand informal carers’ perceptions of the effect of the rehabilitation environment on their family members rehabilitation experience.

## 2 METHOD

A qualitative investigation using a phenomenological approach was used to explore the perspective of those patients and informal carers who had experienced rehabilitation in the various facilities. Interviews were conducted with patients and past patients (n = 54), informal carers (n = 23) and rehabilitation staff (n = 90) between 7.4.17 and 14.9.17 from all 3 South Australia metropolitan health networks until data saturation had occurred (see Table 1 for participant characteristics). Patient and informal carer interviews (lasting between 20 and 45 minutes) were undertaken with one experienced female researcher (MK, AMc or AW) or one experienced male consumer advocate (DF, AP or PH). All staff focus groups (which lasted between 45 and 75 minutes) were conducted by MK with one consumer (DF, AP or PH) assisting with running the group. Prior to commencement of data collection, all investigators collaborated to develop the interview guides (see Appendices [Link], [Link], [Link]) and each person observed at least one interview with the experienced qualitative interviewer (MK) to ensure fidelity and consistency of approach as well as providing opportunities for training. The three facilities were all configured differently. One facility (Facility A) provided all inpatient rehabilitation on the third and fourth floors of a refurbished multi‐storey rehabilitation building, another provided rehabilitation solely in ground floor buildings (Facility B), and the third rehabilitation service provided rehabilitation in two ground floor wards and one ward located on the second floor of a multi‐storey building (Facility C).

**Table 1:** Participant descriptors

Facility	Facility A	Facility B	Facility C
Patients			
Total	15	24	15
Gender (female)	6	10	8
Carer also interviewed (separately)	8	9	4
Informal carers			
Total	8	22	6
Gender (female)	7	8	5
Patient also interviewed (separately)	8	9	4
Facility staff			
Total staff	16	52	22
Total focus groups	6	9	6
Nursing staff^a^	6	35	5
Allied Health^b^	8	16	12
Rehabilitation physicians	1	0	4
Service managers	1	1	1

a Nursing staff consisted of Enrolled Nurses, Registered Nurses, Nurse Unit Managers and Associate Nurse Unit Managers.

b Allied Health consisted of Speech Pathologists, Dieticians, Social Workers, Exercise Physiologists, Physiotherapists, Occupational Therapists.

Clinical staff from each rehabilitation facility were provided information on the study trial and asked to refer patients, patients who had been admitted within the last 6 months, and informal carers if they agreed to being contacted by research staff on the ward or by phone, if at home, to explain the trial and make a time to meet. Clinical staff and managers were also invited (by email) to contact the researchers if they were interested in participating in staff focus groups. As only those who showed an interest in being interviewed were referred to the researchers, very few people decided not to participate once approached (two patients who felt it would take too much time and 1 informal carer who became disinterested in undertaking an interview). Each interview was audiotaped and transcribed verbatim. Staff focus groups were undertaken in the three rehabilitation services according to the convenience of participants. An interview guide was followed to ensure questions asked were related to the topic, but the sequence of questions differed for each participant and offered flexibility so that each participant could discuss any issue that were relevant to their situation (see Appendices [Link], [Link], [Link]: Interview Guides).

To ensure credibility that a comprehensive report was completed prior to analysis, the researchers continued to seek information until all insights and understandings had been gained from the participants at interview.^20^, ^21^ Questions were reframed to gain more information or clarification sought.^21^ Dependability and credibility were enhanced by keeping a journal and audit trail during the research.

### 2.1 Data analysis

Transcriptions were sent to participants for member checking before thematic analysis using NVivo 11 qualitative software. No repeat interviews were required, and participants only made minor changes to the transcripts which were altered before analyses. The data underwent open, axial and selective coding by two researchers (MK and DF). Two health consumer representatives (DF and AP) met with MK and MC on two occasions to develop the draft themes. Subsequently, MK and DF met on another three occasions to review the data and derive the final themes (see Appendix S4: Coding Tree).

## 3 RESULTS

Qualitative analysis resulted in four major themes; (a) choice can be an Illusion in a rehabilitation ward; (b) access to outside areas is a priority and affects well‐being; (c) socialization can be facilitated by the environment; and (d) Ward configuration should align with the model of care.

### 3.1 Choice can be an Illusion in a rehabilitation ward

Most people interviewed reported overall satisfaction with their environment initially and were somewhat perplexed when asked probing questions relating to choices and freedom to undertake meaningful activities and enjoy aspects of their environment. Many patients felt that they were on the ward to undertake their rehabilitation sessions only, and accepted a restrictive environment spending time either in the room where they slept or the therapy room. However, after probing and further questioning, it appeared that many patients, provided the opportunity, would like to make choices about what to do and where to go, in addition to undertaking the tasks or rehabilitation they needed. It was those participants who enjoyed the most choices within their environment already who were the most likely to speak enthusiastically about this issue and to provide even more options that could improve their rehabilitation journey. They wanted to be familiar with the ward environment and be able to utilize areas of the facility that enabled them to carry on with their life to their capacity. Patients wanted to access facilities inside like the therapy gym and kitchen area either independently when able or with their family, and they were keen to spend time outside with visiting family, and friends, and for suitable spaces be available to meet with their pets and allow children to be entertained while staying safe.

That's the first thing I commented on that I didn't like here was the fact that there was nowhere you could go and make yourself a drink in between meals.(Facility B; Patient)

I can understand [the kiosk] it not being open of a weekend because it's run by volunteers, but I wish it was. I'm being selfish.(Facility B; Patient)

Those participants who were restricted to a smaller life space diameter due to the geography of the ward, with few choices, were often quite accepting of their situation. However, those same people were critical if they were unfamiliar with the available facilities in an already diminished environment. A family member said

….there was a few times when I wanted to go in to the dining room, just to sit down and have a coffee with Mum and that, but I didn't feel that I had that right to do that. Because nobody said anything to me, I thought that was just for patients. (Facility A; Informal carer)

In addition to the physical barriers identified, a number of patients felt frustration at their inability to maintain contact with family and their community (including banks, businesses) due to lack of WiFi access, and poor connectivity for mobile phones. They were keen that rehabilitation facilities include these issues in future planning.

### 3.2 Access to outside areas is a priority and affects well‐being

This issue was addressed differently depending on the environment the rehabilitation service patients and carers utilized. All patients and carers interviewed at Facility B discussed the issue passionately, as they reflected on the easy access to outside, the variety of beautiful gardens and courtyards, and the option to utilize undercover areas when they wished. All patients and carers valued the ground floor accommodation, which allowed frequent opportunities to go outside, for them to meet in private with family and to spend time with their precious pets. Access was not restricted for patients, with reports of staff helping patients to get outside and sit in nearby courtyards which were visible from the ward or near the ward. The language used by those being interviewed was colourful and rich as they described the environment which they valued.

In contrast, patients and informal carers from Facility A were not as enthusiastic, even though they still voiced a preference for easy access outside and bemoaned their inability to access outside, which was only available to them when family visited. Of the 15 patients interviewed at Facility A, only one patient reported that they had been assisted to get outside by a staff member on one occasion when they had asked for assistance (Facility A, Patient 10). All the other patients interviewed at Facility A were only able to leave the ward infrequently with the assistance of family (Facility A, Patients 2, 3, 4, 5, 6, 7, 8, 9 and 10). Staff interviewed at Facility C were able to compare easy outside ground floor access for patients from two rehabilitation wards to access by lift for patients accommodated on the second floor of the third ward. However, the enthusiasm for easy access outside was dampened at Facility C as the outdoor spaces were unkempt as a decision had been made to move the hospital in the near future. The patients and carers noticed this and were less keen to utilize the outdoor spaces. Many patients from Facility C also discussed the need for undercover spaces to provide shade in summer and shelter in winter to enable outside access as outside spaces were unprotected from the weather. The ability to get away from the clinical setting was important to many patients and offered improved well‐being

.…gardens at […rehabilitation facility] because they are magnificent and they were a real joy and very healing and every person in my ward—and I was the longest one there so I've had a lot of people going through and every one appreciated all of that.(Facility B; Patient)

There were some specific recommendations made by people interviewed including access to outside for those with severe mobility limitations and those with cognitive impairments, as well as space suitable for different cohorts including Aboriginal and Torres Strait Islander patients. In addition, patients, family and staff emphasized the importance of visiting families, children and pets being able to meet with rehabilitation patients away from the clinical setting in pleasant outdoor surroundings and, in particular, to celebrate significant life milestones.

Some of the quads [people with quadriplegia] we've got in now, they can't do anything but move their heads. They are able to go outside by themselves, and if they were up on the fifth floor or somewhere they won't be able to do that.(Facility B; Rehabilitation clinician)

…. on BIRU [Brain Injury Rehabilitation Unit] the secure garden flows from our ward, so confused patients can still find their way out there even if they're not orientated to the ward, so have an opportunity to go outside and enjoy fresh air. Whereas, if it's multi‐storey I can't see our patients problem‐solving a lift.(Facility B; Rehabilitation clinician)

Actually I think that [Aboriginal patients] often made the most comments about what a relief it is just to get out to where they can sit on the grass or not feel like they're stuck inside, get fresh air ……..just that's been their usual place is outside under this tree on the grass.(Facility B; Rehabilitation clinician)

Something where family members can actually go and just spend a bit of time on their own, but we just don't have the space.(Facility A; Rehabilitation clinician)

### 3.3 Socialization can be facilitated by the environment

It was clear from patients’ comments that not all rehabilitation facilities encourage socialization or even provide areas away from sleeping areas where people can meet. Although the majority of participants interviewed preferred single room accommodation (65%), a number of people were willing to forgo the privacy of a single room to reduce their sense of isolation and loneliness. Forty‐six percentage of patients at Facility A, 21% of patients at Facility B and 13% of patients as Facility C preferred shared room accommodation. Facility A did not have a lounge or sitting room on the ward, while Facility B and Facility C provided one. Patients suggested that if we are to provide single rooms for them, rehabilitation facilities must work to promote opportunities and spaces for patients to meet with other patients, to relax in a social environment and to spend time with visiting family and friends.

There wasn't much socialisation going on, really. No. There were nurses, but no, not talking to others, no.(Facility B; Patient)

You've got the quietness of having a private room, a single room. I suppose the benefits are from having three or four people in a room, you've got company sort of thing and that helps…..that would help and make you feel a bit better too, I suppose, get your mind off things.(Facility B; Patient)

Families receiving support from other patients and their loved ones was another reason patients were keen for rehabilitation facilities to enable socialization.

… it gives them [family members] a better understanding that I'm not the only one in this position ……That peer support…(Facility A; Patient)

Participants reported that multiple socialization and leisure spaces should be provided to afford patients choices.

Unless it's their choice, I think that's where the multiple spaces is important because some people might be going through something life‐changing.……… in some ways I think we need to protect them a little bit as well……. having smaller spaces where it's not so overwhelming and so in your face and where we don't force them to socialise I think is important.(Facility B; Rehabilitation clinician)

### 3.4 Ward configuration should align with the model of care

On delving into patients and families experiences, a number of consumers suggested that their rehabilitation environment did not appear to be person‐ and family‐centred, with few design features that encouraged activity, socialization and family engagement, particularly with visiting children, outside of therapy sessions. The exception to these perceptions was when patients and family had easy and frequent access to outside, and when suitable relaxation, leisure and activity areas were available and accessible. In addition, people who were more dependant reported less choice and opportunities than those who were more mobile, usually only attending therapy sessions and then returning to their bedroom accommodation. However, even those participants who were more ambulatory often described a very sedentary and repetitive rehabilitation day with limited appealing opportunities for inside and outside activities. In addition, patients talked about waiting for access to an insufficient number of facilities (bathrooms and toilets), which meant they were late for therapies. There seemed to be many barriers, both geographical and attitudinal, to patients engaging in valued activities on the ward and, for many patients, few prospects of leaving the clinical area.

But, to me, if you are trying to get people back into the normality of life, then what should be being provided in the rehab facilities are facilities that are as “normal” as possible, and allowing people the opportunity to get out, take a wheelchair around the garden, but also maybe handrails or something there so that you're not just restricted to going to the rehab—whatever they call it—gym. But rather you can get out, you can have a bit of fresh air, you can—I know there's all sorts of restrictions on what you could and couldn't do with that. But, to me, getting people into the frame of mind of normality as soon as possible is a highly valuable part of the physiotherapy process.(Facility A; Patient)

In addition, stimulation and activity should be provided and evident to patients. Participants suggested that activity spaces within the rehabilitation facility were central to the ward and easily accessible to all patients.

I think the biggest thing has been having for people with brain injury, having things all together having the communal spaces adjacent to the bedrooms and the dining room and that sort of thing. So some people who are, they're right there and they have the chance to see or join in, those who wouldn't be able to safely walk to one of their own, can get there because it's next door.(Facility B; Rehabilitation clinician)

Many participants discussed lack of access to spaces and facilities they would enjoy due to explanations they had been provided relating to clinicians’ duty of care. However, a number of clinicians strongly agreed that the risk‐adverse approach to therapy space access was inappropriate.

I feel that in rehab, we're promoting independence but a lot of what we do is making people a little bit dependent.(Facility C; Rehabilitation clinician)

## 4 DISCUSSION

Steven Paul Jobs the American business magnate and investor and co‐founder of Apple Inc. shared, “I think Henry Ford once said, ‘If I'd asked customers what they wanted, they would have told me, “A faster horse!”’ People don't know what they want until you show it to them”.^22^ Ford's famous quote resonated in this current trial as participants provided varying responses and levels of emotion dependant on which rehabilitation facility they had spent time in, and the number of affordances they were provided within their rehabilitation facility. Those people who enjoyed the most choice and access to facilities showed the greatest enthusiasm for these affordances, while those who did not have similar access were more accepting and less concerned about a more restrictive environment until probed to consider the possibilities. In addition, Ford's “Any colour…so long as it is black” approach^23^ might be a philosophy designers of health facilities could dangerously adopt as this current study suggests that many patients and families will accept this restrictive approach if they are not aware of alternatives.

When designing a rehabilitation building, building codes and standards guide construction in relation to air quality, building materials, water supply and so on. However, there remains a gap in addressing the functional and psychological impact of design aspects on the client in policy making and codes during the planning and development process, which is contradiction to Ulrich's proposal of a stress‐free environment.^14^, ^24^ Human habitats can provide environments that facilitate both physical and emotional responses in people who come in contact with them,^25^ and it seems likely that a human body will react to the environment both consciously and subconsciously continually.^26^ This concept has been previously described^16^ and is supported by the findings of this current study.

Parallel to research exploring opportunities for increased physical activity within general rehabilitation environments, there is now emerging work investigating methods of affecting psycho‐social aspects.^6^ The built environment can play a role in psycho‐environmental dynamics and reflects on research that has shown the environment to have a significant effect on the psychogenesis of mental illness.^6^ As psycho‐social aspects of recovery after general rehabilitation are also important, it seems vital to consider these findings in this additional context. Participants in this current study were able to suggest many affordances to improve their feelings of well‐being, and most of these suggestions related to issues aside from their formal therapy sessions. Clearly, the quality of time spent outside formal therapy sessions needs to become a higher priority for designers of rehabilitation facilities.

A systematic review of qualitative studies confirmed the importance and relevance to consumers of a number of the findings in this current study including the importance of offering options and choice to patients undertaking rehabilitation.^27^ This current study suggested that the rehabilitation environment could influence the level of control assumed by patients undertaking rehabilitation by supporting patients and family members to be oriented to their environment, and allowing easy access to those areas of the environment that were important to them. Independent exercise areas, areas to socialize with family and other patients and easy access to outside were core requirements reported by participants. A number of studies have suggested that “when the environment and person act on each other in a consistent and equivalent way” there is a positive impact on health and well‐being.^25^, ^28^, ^29^ To achieve this, “empathic design” architects need to understand users’ needs and preferences regarding what they want to do and how they wish to engage.^30^

It is acknowledged that psychological comfort of patients’ undertaking rehabilitation is important for good outcomes.^14^, ^31^ Participants highlighted similar positive attributes as other studies undertaken in hospitals, including clear signage and other way finding measures, proximity of family members and pleasant décor.^32^ This study undertaken in three large rehabilitation services in South Australia emphasized additional aspects including easy access to outside and environmental aspects that promote choice in the manner in which they socialize and spend their time outside of formal therapy sessions. The research has demonstrated that many assumptions designers make may not be valid, including limiting a rehabilitation environment to sleeping and therapy spaces and expecting that this restrictive environment can support best practice rehabilitation.

Despite an attempt for health services to modernize buildings and engage sophisticated technology, users report that they can find these facilities more confusing than old buildings with poorer facilities.^32^ Although it is inevitable that facilities need to be superseded by new, smarter buildings, it will be important to consider the end users’ satisfaction in such automated facilities. If buildings do, in fact, influence a patient's physical and psychological resources, it is important to ensure the environment eliminates “noxious” stimuli including loss of identity, loss of loved ones, inability to control their immediate environment, disruption of community life and lack of privacy.^33^ Heft^34^ points out that the stimuli of “misaffordance” results in psychological discomfort when users of services are unable to easily gauge functional properties of a building or space, and this subsequently affects patients’ attitude and behaviour through the processes of sensation, perception and cognition.^35^

Patients in other similar studies described feelings of disempowerment and subordination and feeling under “guardianship”.^36^ This current study suggests that these feelings can be ameliorated when the environment supports people to freely utilize the facilities and offers active involvement in activities that they value. Other studies also support the importance of providing opportunities for independent activities as a way of increasing patient control and promoting feelings of positivity and well‐being.^37^, ^38^ Similar to this current study, another qualitative systematic review reported carers’ distress as they “navigated a foreign culture and environment” and the importance of providing a more inclusive milieu as they prepared to assume a caregiver or supportive role.^39^

The importance of access to nature was a repetitive theme amongst interviewed participants who reported the relief of being able to get away from the clinical environment, and the pressures and expectations that could pose. It helps to understand patients’ feelings about being outside when we consider the Attention Restoration Theory posed by Rachel and Stephen Kaplan in 1989.^40^ They proposed that nature can replenish a person's mental and attentional capacity by helping people recover from directed attention fatigue, which frequently occurs in response to attending rehabilitation sessions.^41^ In addition, a number of studies demonstrate that the majority of people prefer natural landscapes to urban views and this is most pronounced when the urban scenes lack vegetation and water features.^31^

There was a strong preference in this study that the rehabilitation environment be suitable to support patients’ and families’ needs, with a compelling argument to provide both indoor and outdoor safe activities for visiting children, to satisfy family re‐engagement goals and socialization needs, and this has been reinforced in other studies.^42^

Participants in this current study expanded on their preferences by suggesting that inside spaces should be configured variously to enable socialization and leisure activities to reflect patient and family likes and needs. Many interviewees emphasized the need to provide the option of open plan, central and visible areas to encourage engagement as well as discreet, private areas for quiet time to cater for different personalities and needs. Research investigating the socio‐physical environment afforded by open plan offices has shown that this environment can nurture or hinder interactive opportunities depending on how each individual perceives the space, and the same space can result in anxiety in some people and feelings of well‐being in others.^43^ Clearly, designers of rehabilitation facilities should consider the different mixes of patients and families who are likely to use the facility and reject the “one size fits all” approach.

The current study clearly emphasizes the importance of patients being able to escape the clinical environment and to socialize with other patients and family visitors to prevent loneliness and boredom when formal therapy sessions are not occurring. Patients in another study agreed with these findings and commented on boredom and lack of stimulation which impacted on their morale and impacted on their progress in rehabilitation.^42^ In addition, as in our current study, other research has indicated that providing areas where patients can meet together and discuss their various rehabilitation journeys allows a culture of motivational peer support and helps maintain their energy and interest in the rehabilitation process.^44^

Other research has shown the many benefits of allowing patients access to their pets while they are in hospital including physical and psychological.^45^ This current research also indicates that patients require somewhere suitable to meet with their visiting pets to reduce their anxiety and lift their mood.

This research provides the consumer perspective of the critical requirements to consider when designing an inpatient rehabilitation facility to facilitate an optimum rehabilitation model of care (see Table 2 for summary of recommendations). Patients and informal carers were keen to share their perspectives and did so with a deal of clarity and provided examples to explain their viewpoint. However, there was a sense of acceptance when patients and family were interviewed in those environments, which offered less choice, less access and less opportunity to undertake activities they valued and the interviewer needed to provide sensitive probing to understand their perspectives, without influencing their answers. This was in contrast to those people who had experienced a more enriched environment who were more forthcoming and easily produced rich and in‐depth perspectives. Whether this matters would depend on studies which include assessment of clinical outcomes. Future work should include measures of quality of life and recovery.

**Table 2:** Design recommendations arising out of consumer interviews

Indoor spaces
Mix of single and shared sleeping accommodation
Therapy gymnasium central to ward and open for use by patients outside therapy times
Kitchen facilities usable for patients and family members
Child friendly area that is safe and entertaining
Various accessible areas to spend time away from sleeping accommodation; designed to provide mix of calm and stimulating opportunities
WiFi connectivity to allow communication with home and community
Clear signage and orientation to ensure patients and informal carers are aware of accessible areas to utilize
Outdoor spaces
Easy access to outside for all patients regardless of their burden of care; need to consider those patients with marked physical, behavioural and emotional challenges
Outside areas close to ward that can be monitored by staff or electronic monitoring systems
Child safe areas outside for visiting children
Areas suitable for visiting pets
Undercover and protected areas to provide usable space all year round
Wide open spaces and accessible pathways to allow access for wheelchair users
Well‐maintained gardens to encourage use
Grass areas and trees for Aboriginal and Torres Strait Islander patients and their visitors to utilize
Kiosk or Café accessible from ward and open after hours to provide socialization opportunities away from the clinical area

## CONFLICT OF INTEREST

Dr Maggie Killington, Mr Dean Fyfe, Mr Allan Patching, Mr Paul Habib, Dr Annabel McNamara, Ms Rachael Kay, Dr Venugopal Kochiyil and Professor Maria Crotty have no conflicts of interest to declare.
