# Supplementary material for: Rehabilitation environments: Service users’ perspective
Source: Health Expect. 2019 Jan 10;22(3):396–404. doi: 10.1111/hex.12859 (PMC6543154; doi:10.1111/hex.12859)
Supplement: Supplementary file 1 [file HEX-22-396-s001.docx]

**Interview Guide patients/past patients**

We are investigating the rehabilitation environment you are currently in or have recently left and want your opinion regarding the environment in supporting your rehabilitation goals.

1. A number of environmental researchers have suggested that the building or architecture can have a profound impact on the way a patient engages in rehabilitation. Do you support this view ?

If “yes” what do/did you feel were important aspects of the environment that support/supported you in your recovery?

1. Do you think there are aspects of the environment that encouraged you to be more physically active?

Do you think there are aspects that made it harder for you to be more physically active?

1. Do you think there are aspects of the environment that allowed you to socialise when you wished?

Do you think that there were aspects that made it harder for you to socialise when you wished?

1. Do you think there are aspects of the environment that made you just feel better about things? Made you happy?

Do you think there were aspects that made you feel worse about things? Made you feel miserable?

1. Do you think there are aspects of the environment that made it easier for you to get ready to go home?

Do you think that there were aspects that made it harder for you to go home?

1. What is good about this current design and why?
2. What needs to change with this current design and why?
3. Some research has been undertaken that discusses design imperatives in psychiatric rehabilitation facilities. These authors have described components that they feel should be considered. Do you consider any of these aspects important in this rehabilitation facility?
4. COMPLEXITY: How “busy” was the environment? Was there much happening? Was it noisy/quite? Did the level of stimulation suit you? If Yes, why? If No, why not?
5. DISCOVERY: Did you feel motivated to explore the ward/area? Was it an interesting place? If yes, why? If Not why not?
6. CONNECTION: Did you feel a connection to the ward/unit/area? If Yes, what sort of connection? If No, what would you have needed to change to feel a connection?
7. CONTROL: Do you feel that you had any influence over what was happening or the way things were being done? Did you feel that you could manipulate your environment at all?
