# Supplementary material for: Rehabilitation environments: Service users’ perspective
Source: Health Expect. 2019 Jan 10;22(3):396–404. doi: 10.1111/hex.12859 (PMC6543154; doi:10.1111/hex.12859)
Supplement: Supplementary file 2 [file HEX-22-396-s002.docx]

**Interview Guide informal carer/next of kin**

We are investigating the rehabilitation environment your family member is currently in or has recently left and want your opinion regarding the environment in supporting their rehabilitation goals.

1. A number of environmental researchers have suggested that the building or architecture can have a profound impact on the way a patient engages in rehabilitation. Do you support this view ?

If “yes” what do/did you feel were important aspects of the environment that support/supported you in your recovery?

1. Do you think there are aspects of the environment that encouraged your relative to be more physically active?

Do you think that there are aspects that made it harder for your relative to be more physically active?

1. Do you think there are aspects of the environment that allowed your relative to socialise when they wished?

Do you think there are aspects that made it harder for your relative to socialise when they wished?

1. Do you think there are aspects of the environment that made your relative just feel better about things? Made them happy?

Do you think that there are aspects that made your relative feel worse about things? Made them feel unhappy/forlorn?

1. Do you think there are aspects of the environment that made it easier for your relative to get ready to go home?

Do you think that there are aspects that made it harder for your relative to get ready to go home?

1. What is good about this current design and why?
2. What needs to change with this current design and why?
3. Some research has been undertaken that discusses design imperatives in psychiatric rehabilitation facilities. These authors have described components that they feel should be considered. Do you consider any of these aspects important in this rehabilitation facility?
4. COMPLEXITY: How “busy” was the environment? Was there much happening? Was it noisy/quite? Did the level of stimulation suit your relative? If Yes, why? If No, why not?
5. DISCOVERY: Did your relative feel motivated to explore the ward/area? Was it an interesting place? If yes, why? If Not why not?
6. CONNECTION: Did your relative feel a connection to the ward/unit/area? If Yes, what sort of connection? If No, what would you have needed to change for him/her to feel a connection?
7. CONTROL: Do you feel that your relative had any influence over what was happening or the way things were being done? Did you feel that he/she could manipulate his/her environment at all?
8. How did the rehabilitation environment enhance your experience as a regular visitor to the unit? Did you feel comfortable in the unit? What might have made it better?
