# Supplementary material for: Rehabilitation environments: Service users’ perspective
Source: Health Expect. 2019 Jan 10;22(3):396–404. doi: 10.1111/hex.12859 (PMC6543154; doi:10.1111/hex.12859)
Supplement: Supplementary file 3 [file HEX-22-396-s003.docx]

**Interview Guide Hospital Staff (Clinicians)**

We are investigating the rehabilitation environment you currently work in and want your opinion regarding the nature and suitability of the unit/service to meet patient, staff and service goals. We would like you to reflect on the feedback you have received from patients, past patients and informal carers during the time you have worked here.

1. A number of environmental researchers have suggested that the building or architecture can have a profound impact on the way a patient engages in rehabilitation. Do you support this view ? If “yes” what do you see as key elements of the building and overall environment that may have an impact on patient outcomes?
2. Do you think there are aspects of the environment that improve the physical activity levels of patients?

Do you think there are aspects that make it harder for patients to be more physically active?

1. Do you think there are aspects of the environment that improve the ability for patients to socialise more?

Do you think there are aspects that make it harder for patients to socialise more?

1. Do you think there are aspects of the environment that improve patients’ emotional state?

Do you think that there are aspects that worsen patients’ emotional state?

1. Do you think there are aspects of the environment which encourage patients to transfer successfully home?

Do you think that there are aspects that make it harder for patients to transfer successfully home?

1. What is good about this current design and why?
2. What needs to change with this current design and why?
3. Some research has been undertaken that discusses design imperatives in psychiatric rehabilitation facilities. These authors have described components that they feel should be considered. Do you consider any of these aspects important in this rehabilitation facility?
4. COMPLEXITY: What is the level of complexity or the level of arousal that this rehabilitation facility promotes? Do you think it is suitable for the patients currently attending the facility? Do you have any suggestions to improve this component?
5. DISCOVERY: Do you think that this environment promotes a sense of “discovery” so that patients become inquisitive and seek to explore their environment?
6. CONNECTION: Do you have a sense that patients “connect” with the building or facility?
7. CONTROL: Do you feel that patients have opportunities to manage aspects of their environment according to their wishes and needs?
