# Supplementary material for: Rehabilitation environments: Service users’ perspective
Source: Health Expect. 2019 Jan 10;22(3):396–404. doi: 10.1111/hex.12859 (PMC6543154; doi:10.1111/hex.12859)
Supplement: Supplementary file 4 [file HEX-22-396-s004.docx]

| **Codes** | **Subthemes** | **Themes** |
| --- | --- | --- |
| - Overall satisfaction - Level of affordances offered - Choices provided - Did experience affect perceptions? - Is rehabilitation more than formal therapy sessions? - Patients and families knowing where they can go/what they can do - Kiosk/café availability (also after hours) important - Feeling in control - Getting outside often not possible - Activity is facilitated if seeing and therefore knowing what is on - Being able to use facilities independently when able - Hurried admissions increase discomfort - Waiting - Availability of equipment eg wheelchairs to go out, enough bathrooms | - Those who had restrictive environment accepting of situation until probed - Those with more opportunities provided expected and valued these affordances - Thought rehabilitation was just therapy sessions - Patient and family orientation to ward should be a priority - Reduced access to outdoors reduced choices - Activity/therapy areas provided in sight of patients; if you cant see it….dont know about it - Easy access to ward gym and kitchen out of hours - Provision of enough resources eg bathrooms to eliminate excessive waiting - Opportunities for patients and families to engage in activities safely | **Choice can be an illusion in a rehabilitation ward** |
| - Getting outside provides relief from challenges of rehabilitation - Getting outside increases wellbeing - Accessible and attractive gardens improve morale - Multi-story buildings barrier to getting outside - Weekend activity/therapy eg kiosk, places to sit, things to do - Family time outside reduces stress - Community facilities on site - Outside therapy areas are fun - Patients with high burden of care (physical) - Patients with high burden of care (behavioural) - Patients with cultural needs to access outside - factors to consider when designing outside space | - Getting outside and away from clinical setting is important - Barriers to getting outside greater when multi-story - Staff not able to provide outside access in multistorey facility - Lifts not able to be used by most patients who are challenged physically, behaviouraly and cognitively - Outside space for families especially children - Outside space to allow pets to visit - Outside space as a therapy resource | **Access to outside areas is a priority and affects well-being** |
| - Very few opportunities to meet other patients - Shared bedrooms only way to meet people - Prefer single room but lonely - Family areas important - Different social areas important - Good to be able to compare progress and rehabilitation journey with others - Social areas need to be appealing before patients will use them - Computer/internet access/TV | - If single rooms are provided need to ensure socialisation opportunities - Need to provide different lounge areas to suit various needs - Social areas need to be attractive - Space suitable for children inside and outside allows parents and grandparents to continue their important roles/interaction with children - Good internet access, computer access and phone connectivity should be provided to support patient contact with their community | **Socialisation can be facilitated by the environment.** |
| - Rehabilitation needs to prepare for return to community - Patient equity - Rehabilitation more than just therapy - Rehabilitation wards need to be set up to encourage patient and family engagement - Excessive risk aversion can reduce patient independence - Facilities need to enhance patient autonomy - Facilities need to facilitate opportunities to be active - Facilities need to facilitate opportunities for socialisation and cognitive challenges - Community facilities on site support transition home - Therapy areas need to be accessible - Transition home needs to be target when designing facility - Ward configuration is often not conducive to supporting rehabilitation goals - Weekend therapy and activity to reduce boredom | - Family friendly areas inside and outside - Availability of facilities eg bathrooms to reduce excessive waiting and/or missing therapy - Therapy and activity opportunities to be provided that patients are aware of and accessible for all irrespective of their level of disability - Social areas that encompass different needs; central open areas and quite, smaller areas - Community facilities eg basketball/rugby court on site to allow people (particularly those with spinal cord and brain injury) to engage when still inpatients - Opportunities to access facilities out of hours eg gymnasium, ward kitchen, kiosk - Provide therapy on weekends, and some group activities | **Ward configuration should align with the Model of Care**. |
